# Supplementary material for: Comparative genomics provides new insights into the diversity, physiology, and sexuality of the only industrially exploited tremellomycete: Phaffia rhodozyma
Source: BMC Genomics. 2016 Nov 9;17:901. doi: 10.1186/s12864-016-3244-7 (PMC5103461; doi:10.1186/s12864-016-3244-7)
Supplement: Additional file 6: — List of orphan genes with links to PFAM (related to Additional file 1: Table S1). (ZIP 1428 kb) [file 12864_2016_3244_MOESM6_ESM.zip › PFAM_UNIQUE/G00676_P.html]

#                                                                            --- full sequence --- -------------- this domain -------------   hmm coord   ali coord   env coord
  
# target name        accession   tlen query name           accession   qlen   E-value  score  bias   #  of  c-Evalue  i-Evalue  score  bias  from    to  from    to  from    to  acc description of target
  
#------------------- ---------- ----- -------------------- ---------- ----- --------- ------ ----- --- --- --------- --------- ------ ----- ----- ----- ----- ----- ----- ----- ---- ---------------------
  
G00676\_P             -            311 DUF4402              PF14352.1    130       2.2    7.5   5.2   1   2   0.00029       1.7    7.8   3.0    29    90    16    94     5   112 0.70 - Domain of unknown function (DUF4402)  
G00676\_P             -            311 DUF4402              PF14352.1    130       2.2    7.5   5.2   2   2       0.1     6e+02   -0.5   0.6    27    92   105   169    81   178 0.49 - Domain of unknown function (DUF4402)
